# Supplementary material for: Promoterless gene targeting without nucleases rescues lethality of a Crigler‐Najjar syndrome mouse model
Source: EMBO Mol Med. 2017 Jul 27;9(10):1346–55. doi: 10.15252/emmm.201707601 (PMC5623861; doi:10.15252/emmm.201707601)
Supplement: Supplementary file 1 — Appendix [file EMMM-9-1346-s001.pdf]

Promoterless gene targeting without nucleases rescues lethality of a Crigler-Najjar syndrome mouse model

Fabiola Porro[1,#], Giulia Bortolussi[1,#], Adi Barzel[2]†, Alessia De Caneva[1], Alessandra Iaconcig[1], Simone Vodret[1], Lorena Zentilin[1], Mark A. Kay[2] and Andrés F. Muro[1*]

[1] ICGEB, Trieste, TS, Italy, 34149.

[2] Departments of Pediatrics and Genetics, Stanford University, Stanford, CA, United States.

APPENDIX

Table of contents

Legends to the Appendix Figures

Appendix Figure S1. Western blot analysis of Ugt1$^{-/-}$ mice treated with the rAAV-Alb-hUGT1a1 donor vector.

Appendix Figure S2. Analysis of inflammatory markers in rAAV-Alb-hUGT1a1 treated mice.

Appendix Figure S3. Analysis of inflammatory markers in WT mice treated with the rAAV-Alb-eGFP donor vector at different administration days, doses and modalities.

Appendix Table S1. List of oligonucleotides

Appendix Table S2. Shapiro-Wilk normality test

Legends to the Appendix Figures

Appendix Figure S1. Western blot analysis of Ugt1$^{-/-}$ mice treated with the rAAV8-Alb-hUGT1a1 donor vector.

Ugt1$^{-/-}$ mice injected at P4 (lanes 3-14) and P10 (lanes 15-17) with 1.0E12 vgp/mouse of rAAV-Alb-hUGT1a1 donor vector were sacrificed at 1, 4 and 12 months after viral transfer (1 month: lanes 3-4, 11-12, 15-17; 4 months: lanes 5-7; 12 months: lanes 8-10, 13-14). Liver protein extracts (50 µg) were loaded in a SDS-PAGE, transferred to a nitrocellulose membrane and developed using an antibody anti-Ugt1 (Santa Cruz Biotechnologies). Long and short exposures are shown (Panels A and B, respectively). As controls, we loaded 20 µg of a WT liver (lanes 1 and 20) and 50 µg of untreated mutant liver (lane 19). Actin (indicated by *) was used as control for protein load (Panel C). Lanes 2 and 18, molecular weigh markers. The arrows indicate the Ugt1a1 band (Panels A and B).

Appendix Figure S2. Analysis of inflammatory markers in rAAV-Alb-hUGT1a1 treated mice.

Inflammatory markers (CD8, CD4 and TNFα) were determined by qRT-PCR in total RNA samples from treated animals (P4, 1.0E12 vgp/mouse) sacrificed at 1, 4 and 12 months. Expression levels relative to Gapdh are shown.

Appendix Figure S3. Analysis of inflammatory markers in WT mice treated with the rAAV8-Alb-eGFP donor vector at different administration days, doses and modalities.

A Inflammatory markers (CD8, CD4, TNFα, CD68 and MRC1) were determined by qRT-PCR in total RNA samples from WT animals treated with the rAAV-Alb-eGFP donor vector (0.7E12 and 1.0E12 vgp/mouse) at P2 and P4. Mice were sacrificed at M1 and M4 after viral injection. Expression levels relative to Gapdh are shown. CD8, Two-way ANOVA, Time, P=0.0414; CD4, Two-way ANOVA, Time,

P=0.0219; CD68, Two-way ANOVA, NS; MRC1, Two-way ANOVA, NS; TNFa, Two-way ANOVA, Untr. Vs. P2 1.0E12, $t=2.662$, $P<0.05$.

B Comparison of inflammatory markers (CD8, CD4, TNFα, CD68 and MRC1) in animals transduced with single or multiple injections at P4 with 1.0E12, 2.0E12 or 4.0E12 vgp/mouse of rAAV8-Alb-eGFP donor vector, as described in Fig EV3. Expression levels relative to Gapdh are shown. CD8, One-way ANOVA, *P*=0.0721; CD4, One-way ANOVA, P=0.5595; CD68, One-way ANOVA, *P*=04955; MRC1, One-way ANOVA, *P*=0.9082; TNFa, One-way ANOVA, *P*=0.1778.

Appendix Figure S1

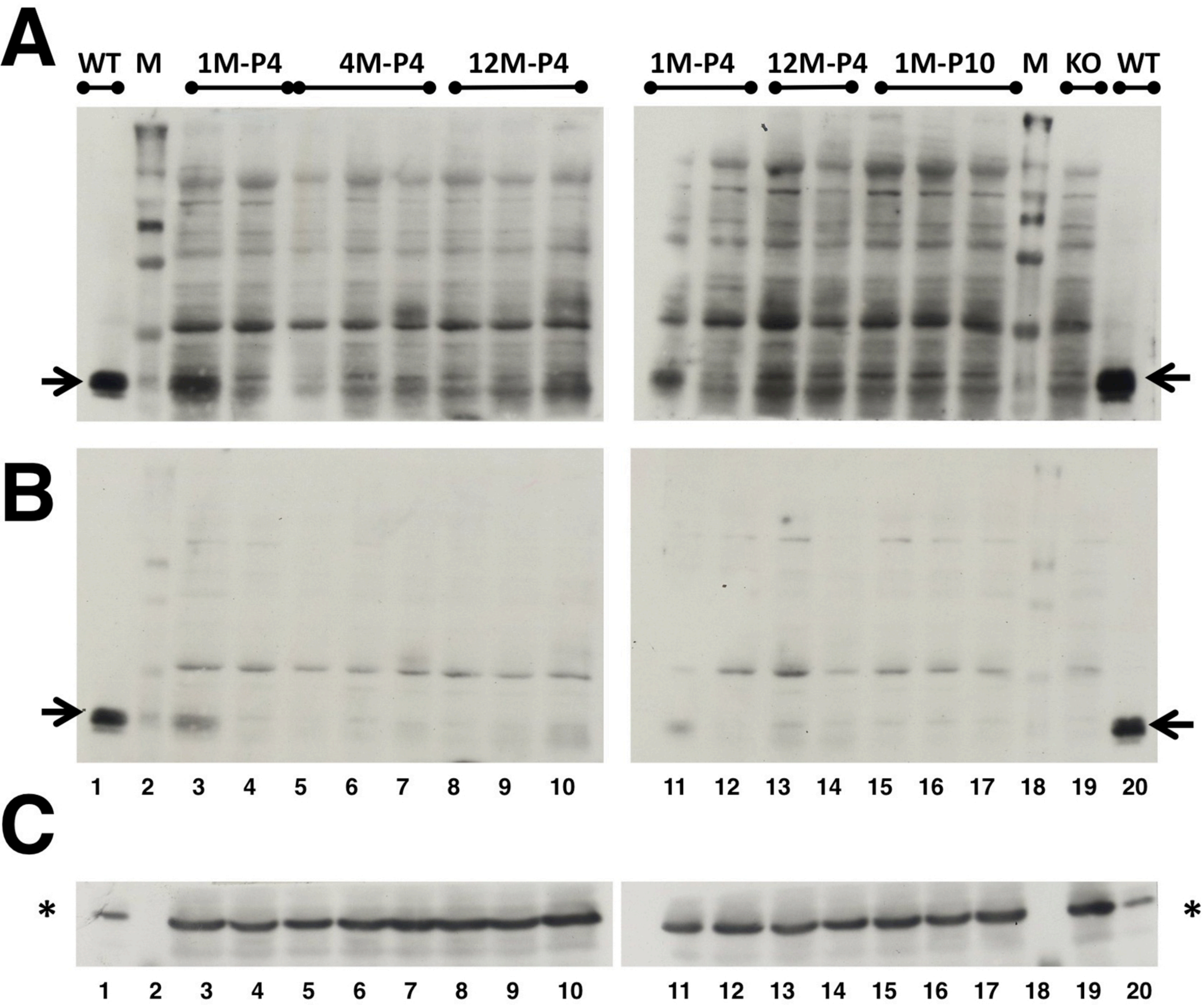

Porro et al., Appendix Figure S1. Western blot analysis of Ugt1$^{-/-}$ mice treated with the rAAV-Alb-hUGT1a1 donor vector. Ugt1$^{-/-}$ mice injected at P4 (lanes 3-14) and P10 (lanes 15-17) with 1.0E12 vgp/mouse of rAAV-Alb-hUGT1a1 donor vector were sacrificed at 1, 4 and 12 months after viral transfer (1 month: lanes 3-4, 11-12, 15-17; 4 months: lanes 5-7; 12 months: lanes 8-10, 13-14). Liver protein extracts (50 μg) were loaded in a SDS-PAGE, transferred to a nitrocellulose membrane and developed using an antibody anti-Ugt1 (Santa Cruz Biotechnologies). Long and short exposures are shown (Panels A and B, respectively). As controls, we loaded 20 μg of a WT liver (lanes 1 and 20) and 50 μg of untreated mutant liver (lane 19). Actin (indicated by *) was used as control for protein load (Panel C). Lanes 2 and 18, molecular weigh markers. The arrows indicate the Ugt1a1 band (Panels A and B).

Appendix Figure S2

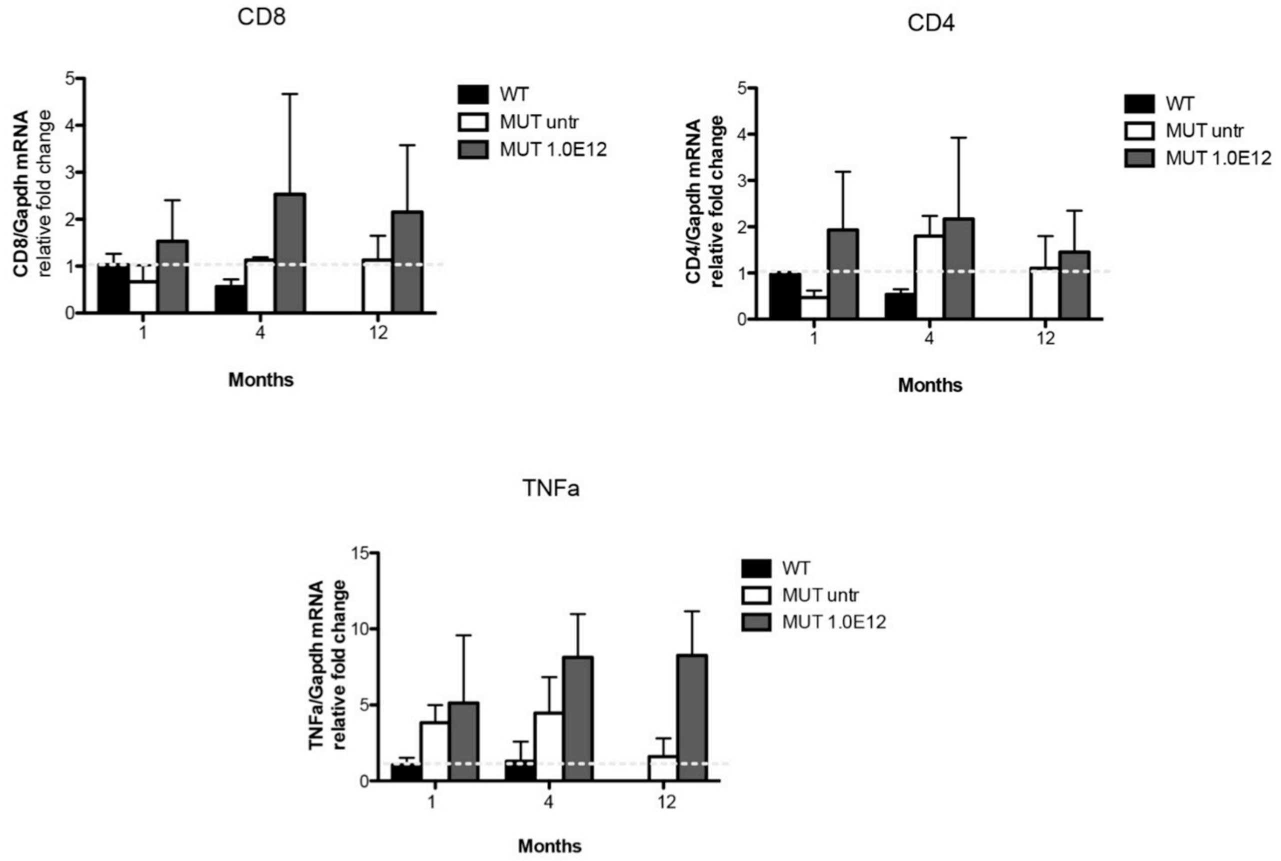

Porro et al., Appendix Figure S2. Analysis of inflammatory markers in rAAV-Alb-hUGT1a1 treated mice. Inflammatory markers (CD8, CD4 and TNFa) were determined by qRT-PCR in total RNA samples from treated animals (P4, 1.0E12 vgp/mouse) sacrificed at 1, 4 and 12 months. Expression levels relative to Gapdh are shown.

Appendix Figure S3

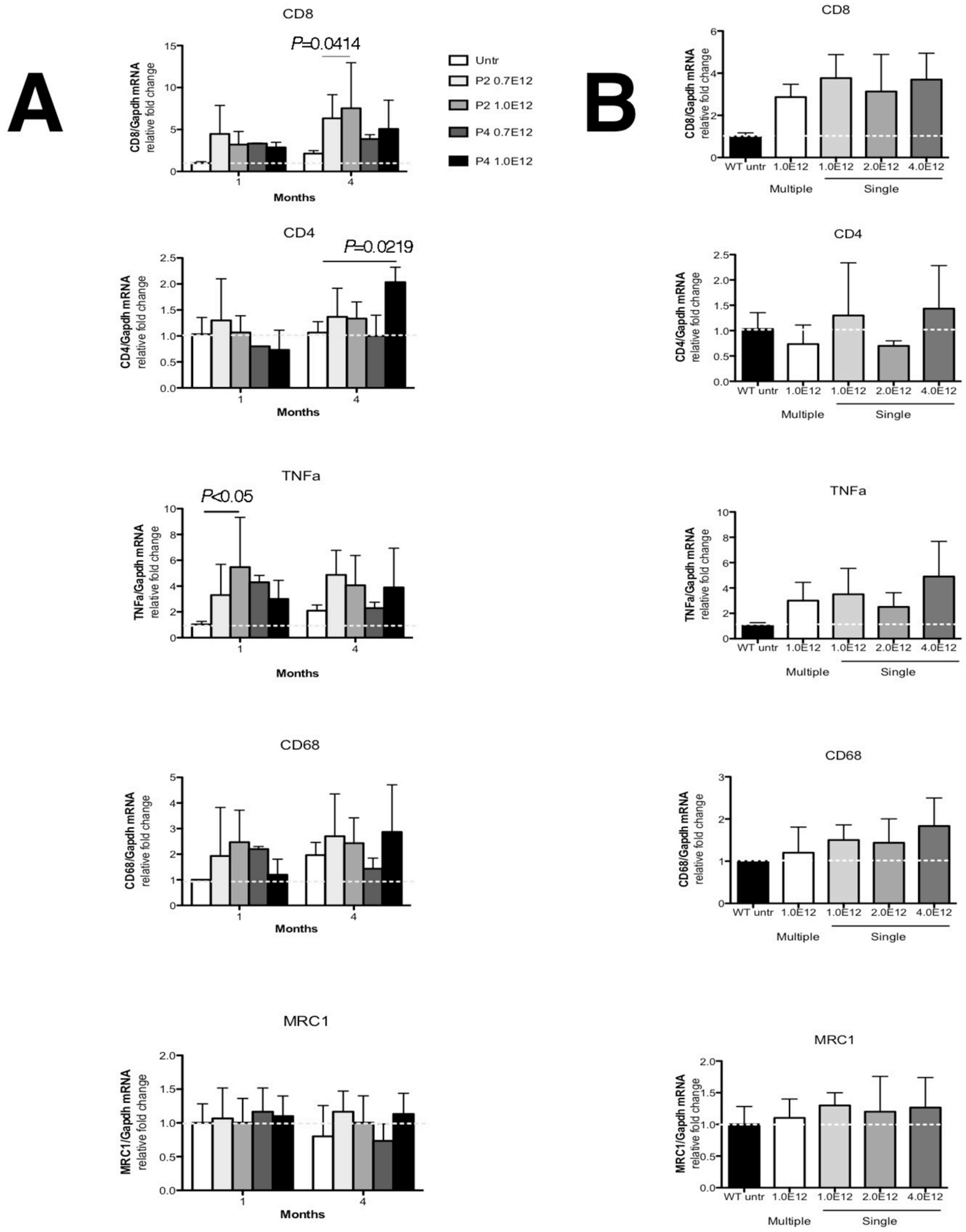

Appendix Figure S3. Analysis of inflammatory markers in WT mice treated with the rAAV-Alb-eGFP donor vector at different administration days, doses and modalities.

A Inflammatory markers (CD8, CD4, TNFa, CD68 and MRC1) were determined by qRT-PCR in total RNA samples from WT animals treated with the rAAV-Alb-eGFP donor vector (0.7E12 and 1.0E12 vgp/mouse) at P2 and P4. Mice were sacrificed at M1 and M4 after viral injection. Expression levels relative to Gapdh are shown. CD8, Two-way ANOVA, Time, P=0.0414; CD4, Two-way ANOVA, Time, P=0.0219; CD68, Two-way ANOVA, NS; MRC1, Two-way ANOVA, NS; TNFa, Two-way ANOVA, Untr. Vs. P2 1.0E12, t=2.662, P<0.05.

B Comparison of inflammatory markers (CD8, CD4, TNFa, CD68 and MRC1) in animals transduced with single or multiple injections at P4 with 1.0E12, 2.0E12 or 4.0E12 vgp/mouse of rAAV-Alb-eGFP donor vector, as described in Fig EV3. Expression levels relative to Gapdh are shown. CD8, One-way ANOVA, P=0.0721; CD4, One-way ANOVA, P=0.5595; CD68, One-way ANOVA, P=04955; MRC1, One-way ANOVA, P=0.9082; TNFa, One-way ANOVA, P=0.1778.

Promoterless gene targeting without nucleases rescues lethality of a Crigler-Najjar syndrome mouse model, by Porro et al.

Appendix Table S1

List of oligonucleotides (5’-3’)

EGFP(BstXI)Dir	CTCCATGGCTGTGGtgagcaagggcgaggagctg
EGFP(NheI) Rev	CAGCTAGCttgtacagctcgtcc atgccgagag
GFP_F	AAG TTC ATC TGC ACC ACC G
GFP For	TGC CCG ACA ACC ACT ACC TG
pAB-EGFP1635Rev	ACC ACC CCG GTG AAC AGC T
mALB_11Rev	TGA GTC CTG AGT CTT CAT GTC TT
mALB_ 10FW	CTG ACA AGG ACA CCT GCT TC
mALB Ex12F1	TGA GAC CTT CAC CTT CCA CTC TG
mALB Ex12F2	CAC ACT TCC AGA GAA GGA GAA GC
hUGT1-191Rev	GCG TCA GGT GCT AGG ACA AC
hUGT1A1-112Rev	GGA TCA ACA GTA TCT TCC CAG C
mUGT1a1dir	TCT GGC TGA TGA GAA GTG ACT
mUGT1a1rev	GAA AAC AAC GAT GCC ATG CT
hUGT1-1471F	GTG ATT GGT TTC CTC TTG GCC GTC GTG
mGAPDH_Dir	GCA TGG ACT GTG GTC ATG AG
mGAPDH_Rev	CCA TCA CCA TCT TCC AGG AG
BstXI –NheI Donor vector linker	CTAGTGCCATGGCTGTGGATATCG
mCD8a DIR	TCAGTTCTGTCGTGCCAGTC
mCD8EX2REV	GCACTGGCTTGGTAGTAGTA

mCD4DIR	GCAGCATGGCAAAGGTGTAT
mCD4REV	AAACGATCAAACTGCGAAGG
mTNFaDIR	TTCGAGTGACAAGCCTGTAG
mTNFaREV	AGACAAGGTACAACCCATCG
mCD68DIR	TACCCAATTCAGGGTGGAAG
mCD68REV	TGTATTCCACCGCCATGTAG
mMRC1DIR	ATTGTGGAGCAGATGGAAGG
mMRC1REV	TTTGTCGTAGTCAGTGGTGG

Porro et al., Appendix Table S2

Shapiro-Wilk Test

Figure	Dataset	Mean	SD	W	N	*P* value
1C	P2 (0.7E12)	0.026	0.003	0.893	3	0.363
1C	P2 (1.0E12)	0.085	0.015	0.996	3	0.886
1C	P4 (0.7E12)	0.035	0.007	0.862	3	0.274
1C	P4 (1.0E12)	0.133	0.023	0.787	3	0.085
1C	P10 (0.7E12)	0.013	0.019	0.773	3	0.051
1C	P10 (1.0E12)	0.036	0.008	0.971	4	0.845
1C	P30 (1.0E12)	0.015	0.002	1.000	3	1.000
1C	P4 (1.0E12)(Ep.)	1.710	0.334	0.976	3	0.702
1C	P30 (1.0E12)(Ep.)	24.470	4.731	0.901	3	0.388
1D	P2 (0.7E12)	9.866	3.659	0.979	3	0.720
1D	P2 (1.0E12)	23.752	7.350	0.906	3	0.405
1D	P4 (0.7E12)	6.241	1.337	0.980	3	0.727
1D	P4 (1.0E12)	57.444	12.351	0.939	3	0.523
1D	P10 (0.7E12)	5.737	7.045	0.830	3	0.187
1D	P10 (1.0E12)	15.928	8.565	0.777	3	0.060
1D	P30 (1.0E12)	17.520	3.603	0.773	3	0.052
2D	WT	151.600	29.300	0.978	13	0.869
2D	WT pAlb-eGFP	158.600	16.800	0.868	5	0.258
2D	Ugt1-/- pAlb-hUgt1a1	158.700	23.700	0.986	5	0.964
2D	Ugt1-/- P8-P20 PT	67.200	46.100	0.971	3	0.674
3E	Ugt1-/- pAlb-hUgt1a1 (1 month)	0.033	0.014	0.835	3	0.202
3E	Ugt1-/- pAlb-hUgt1a1 (12 months)	0.017	0.013	0.899	4	0.426
EV2A	P2 (0.7E12) 1 month	0.026	0.003	0.893	3	0.363
EV2A	P2 (0.7E12) 4 month	0.000	0.000	0.893	3	0.363
EV2A	P2 (1.0E12) 1 month	0.085	0.015	0.996	3	0.886
EV2A	P2 (1.0E12) 4 month	0.010	0.014	0.945	2	0.604
EV2A	P4 (0.7E12) 1 month	0.035	0.007	0.862	3	0.274
EV2A	P4 (0.7E12) 4 month	0.010	0.016	0.776	3	0.058
EV2A	P4 (1.0E12) 1 month	0.133	0.023	0.787	3	0.085
EV2A	P4 (1.0E12) 4 month	0.113	0.102	0.970	3	0.669
EV2C	P4 (1.0E12) Multiple	0.133	0.023	0.787	3	0.085
EV2C	P4 (1.0E12) Single	0.020	0.013	0.953	3	0.583
EV2C	P4 (2.0E12) Single	0.037	0.005	0.832	3	0.194
EV2C	P4 (4.0E12) Single	0.056	0.026	0.969	3	0.664
EV3E	control (PT)	9.540	0.806	0.807	4	0.116
EV3E	P2 (0.7E12)	9.777	1.217	0.776	4	0.065
EV3E	P2 (1.0E12)				1	NA
EV3E	P4 (0.7E12)	4.933	0.702	0.993	3	0.843
EV3E	P4 (1.0E12)	3.350	0.641	0.943	6	0.684
EV3E	P10 (1.0E12)	6.478	1.415	0.937	4	0.637
EV4F	1M	13.580	8.154	0.779	3	0.064
EV4F	4M	7.073	7.979	0.897	3	0.375
EV4F	12M	2.632	1.139	0.880	4	0.339
Appendix Supp. Fig S2-CD8	WT (1 month)	1.033	0.231	0.786	3	0.083
Appendix Supp. Fig S2-CD8	Mut Untr (1 month)	0.667	0.351	0.993	3	0.843
Appendix Supp. Fig S2-CD8	Mut 1.0E12 (1 month)	1.533	0.874	0.947	3	0.554
Appendix Supp. Fig S2-CD8	WT (4 months)	0.567	0.153	0.964	3	0.637
Appendix Supp. Fig S2-CD8	Mut Untr (4 months)	1.133	0.059	0.881	3	0.328
Appendix Supp. Fig S2-CD8	Mut 1.0E12 (4 months)	2.533	2.139	0.789	3	0.089
Appendix Supp. Fig S2-CD8	Mut Untr (12 months)	1.133	0.513	0.949	3	0.567
Appendix Supp. Fig S2-CD8	Mut 1.0E12 (12 months)	2.150	1.427	0.968	4	0.830
Appendix Supp. Fig S2-CD4	WT (1 month)	0.967	0.059	0.881	3	0.328
Appendix Supp. Fig S2-CD4	Mut Untr (1 month)	0.467	0.153	0.964	3	0.637
Appendix Supp. Fig S2-CD4	Mut 1.0E12 (1 month)	1.933	1.258	0.987	3	0.780
Appendix Supp. Fig S2-CD4	WT (4 months)	0.533	0.116	0.821	3	0.165
Appendix Supp. Fig S2-CD4	Mut Untr (4 months)	1.800	0.436	0.842	3	0.220
Appendix Supp. Fig S2-CD4	Mut 1.0E12 (4 months)	2.167	1.762	0.774	3	0.054
Appendix Supp. Fig S2-CD4	Mut Untr (12 months)	0.700	0.490	1.000	3	1.000
Appendix Supp. Fig S2-CD4	Mut 1.0E12 (12 months)	1.450	0.896	0.943	4	0.673
Appendix Supp. Fig S2-TNFa	WT (1 month)	1.067	0.462	0.786	3	0.083
Appendix Supp. Fig S2-TNFa	Mut Untr (1 month)	3.833	1.159	0.821	3	0.165
Appendix Supp. Fig S2-TNFa	Mut 1.0E12 (1 month)	5.133	4.446	0.980	3	0.726
Appendix Supp. Fig S2-TNFa	WT (4 months)	1.300	1.300	0.783	3	0.073
Appendix Supp. Fig S2-TNFa	Mut Untr (4 months)	4.467	2.363	0.907	3	0.407
Appendix Supp. Fig S2-TNFa	Mut 1.0E12 (4 months)	8.133	2.845	0.835	3	0.202
Appendix Supp. Fig S2-TNFa	Mut Untr (12 months)	1.600	1.212	0.980	3	0.726
Appendix Supp. Fig S2-TNFa	Mut 1.0E12 (12 months)	8.250	2.919	0.840	4	0.197
Appendix Supp. Fig S3A-CD8	Untr M1	1.000	0.173	0.798	3	0.110

Appendix Supp. Fig S3A-CD8	P2 0.7E12 M1	4.467	3.408	0.775	3	0.056
Appendix Supp. Fig S3A-CD8	P2 1.0E12 M1	3.200	1.572	0.851	3	0.244
Appendix Supp. Fig S3A-CD8	P4 0.7E12 M1	3.333	0.076	0.964	3	0.637
Appendix Supp. Fig S3A-CD8	P4 1.0E12 M1	2.867	0.611	0.964	3	0.637
Appendix Supp. Fig S3A-CD8	Untr M4	2.133	0.351	0.993	3	0.843
Appendix Supp. Fig S3A-CD8	P2 0.7E12 M4	6.333	2.811	0.823	3	0.170
Appendix Supp. Fig S3A-CD8	P2 1.0E12 M4	7.533	5.424	0.901	3	0.390
Appendix Supp. Fig S3A-CD8	P4 0.7E12 M4	3.867	0.513	0.949	3	0.567
Appendix Supp. Fig S3A-CD8	P4 1.0E12 M4	5.067	3.430	0.844	3	0.223
Appendix Supp. Fig S3A-CD4	Untr M1	1.033	0.321	0.871	3	0.298
Appendix Supp. Fig S3A-CD4	P2 0.7E12 M1	1.300	0.800	1.000	3	1.000
Appendix Supp. Fig S3A-CD4	P2 1.0E12 M1	1.067	0.321	0.871	3	0.298
Appendix Supp. Fig S3A-CD4	P4 0.7E12 M1	0.807	0.015	0.964	3	0.637
Appendix Supp. Fig S3A-CD4	P4 1.0E12 M1	0.733	0.379	0.855	3	0.253
Appendix Supp. Fig S3A-CD4	Untr M4	1.067	0.208	0.923	3	0.463
Appendix Supp. Fig S3A-CD4	P2 0.7E12 M4	1.367	0.551	0.824	3	0.174
Appendix Supp. Fig S3A-CD4	P2 1.0E12 M4	1.333	0.321	0.871	3	0.298
Appendix Supp. Fig S3A-CD4	P4 0.7E12 M4	1.000	0.400	1.000	3	1.000
Appendix Supp. Fig S3A-CD4	P4 1.0E12 M4	2.033	0.306	0.964	3	0.637
Appendix Supp. Fig S3A-TNFa	Untr M1	1.033	0.236	0.907	3	0.472
Appendix Supp. Fig S3A-TNFa	P2 0.7E12 M1	3.300	2.390	0.967	3	0.652
Appendix Supp. Fig S3A-TNFa	P2 1.0E12 M1	5.467	3.859	0.919	3	0.450
Appendix Supp. Fig S3A-TNFa	P4 0.7E12 M1	4.300	0.529	0.893	3	0.363
Appendix Supp. Fig S3A-TNFa	P4 1.0E12 M1	3.000	1.442	0.942	3	0.537
Appendix Supp. Fig S3A-TNFa	Untr M4	2.100	0.436	0.842	3	0.220
Appendix Supp. Fig S3A-TNFa	P2 0.7E12 M4	4.867	1.904	0.996	3	0.885
Appendix Supp. Fig S3A-TNFa	P2 1.0E12 M4	4.067	2.303	0.955	3	0.590
Appendix Supp. Fig S3A-TNFa	P4 0.7E12 M4	2.300	0.458	0.964	3	0.637
Appendix Supp. Fig S3A-TNFa	P4 1.0E12 M4	3.900	3.041	0.818	3	0.157
Appendix Supp. Fig S3A-CD68	Untr M1	1.000	0.141	0.945	2	0.604
Appendix Supp. Fig S3A-CD68	P2 0.7E12 M1	1.933	1.893	0.855	3	0.253
Appendix Supp. Fig S3A-CD68	P2 1.0E12 M1	2.467	1.250	0.846	3	0.229
Appendix Supp. Fig S3A-CD68	P4 0.7E12 M1	2.200	0.100	1.000	3	1.000
Appendix Supp. Fig S3A-CD68	P4 1.0E12 M1	1.200	0.608	0.818	3	0.157
Appendix Supp. Fig S3A-CD68	Untr M4	1.967	0.493	0.832	3	0.194
Appendix Supp. Fig S3A-CD68	P2 0.7E12 M4	2.700	1.652	0.997	3	0.900
Appendix Supp. Fig S3A-CD68	P2 1.0E12 M4	2.433	0.987	0.832	3	0.194
Appendix Supp. Fig S3A-CD68	P4 0.7E12 M4	1.433	0.416	0.923	3	0.463
Appendix Supp. Fig S3A-CD68	P4 1.0E12 M4	2.867	1.845	0.952	3	0.578
Appendix Supp. Fig S3A-MRC1	Untr M1	1.000	0.283	0.945	2	0.604
Appendix Supp. Fig S3A-MRC1	P2 0.7E12 M1	1.067	0.451	0.996	3	0.878
Appendix Supp. Fig S3A-MRC1	P2 1.0E12 M1	1.000	0.361	0.942	3	0.537
Appendix Supp. Fig S3A-MRC1	P4 0.7E12 M1	1.167	0.351	0.993	3	0.843
Appendix Supp. Fig S3A-MRC1	P4 1.0E12 M1	1.100	0.300	1.000	3	1.000
Appendix Supp. Fig S3A-MRC1	Untr M4	0.800	0.458	0.964	3	0.637
Appendix Supp. Fig S3A-MRC1	P2 0.7E12 M4	1.167	0.306	0.964	3	0.637
Appendix Supp. Fig S3A-MRC1	P2 1.0E12 M4	1.000	0.400	1.000	3	1.000
Appendix Supp. Fig S3A-MRC1	P4 0.7E12 M4	0.733	0.252	0.987	3	0.780
Appendix Supp. Fig S3A-MRC1	P4 1.0E12 M4	1.133	0.306	0.964	3	0.637
Appendix Supp. Fig S3B-CD8	WT Untr	1.000	0.180	0.942	3	0.537
Appendix Supp. Fig S3B-CD8	P4 (1.0E12) Multiple	2.867	0.611	0.964	3	0.637
Appendix Supp. Fig S3B-CD8	P4 (1.0E12) Single	3.767	1.115	0.887	3	0.344
Appendix Supp. Fig S3B-CD8	P4 (2.0E12) Single	3.133	1.762	0.774	3	0.054
Appendix Supp. Fig S3B-CD8	P4 (4.0E12) Single					
Appendix Supp. Fig S3B-CD4	WT Untr	1.033	0.321	0.871	3	0.298
Appendix Supp. Fig S3B-CD4	P4 (1.0E12) Multiple	0.733	0.379	0.855	3	0.253
Appendix Supp. Fig S3B-CD4	P4 (1.0E12) Single	1.300	1.044	0.828	3	0.183
Appendix Supp. Fig S3B-CD4	P4 (2.0E12) Single	0.700	0.100	1.000	3	1.000
Appendix Supp. Fig S3B-CD4	P4 (4.0E12) Single	1.433	0.850	0.885	3	0.339
Appendix Supp. Fig S3B-TNFa	WT Untr	1.033	0.252	0.987	3	0.780
Appendix Supp. Fig S3B-TNFa	P4 (1.0E12) Multiple	3.000	1.442	0.942	3	0.537
Appendix Supp. Fig S3B-TNFa	P4 (1.0E12) Single	3.500	2.042	0.912	3	0.424
Appendix Supp. Fig S3B-TNFa	P4 (2.0E12) Single	2.500	1.127	0.787	3	0.085
Appendix Supp. Fig S3B-TNFa	P4 (4.0E12) Single	4.900	2.778	0.810	3	0.138
Appendix Supp. Fig S3B-CD68	WT Untr	1.000	0.000		2	NA
Appendix Supp. Fig S3B-CD68	P4 (1.0E12) Multiple	1.200	0.608	0.818	3	0.157
Appendix Supp. Fig S3B-CD68	P4 (1.0E12) Single	1.500	0.361	0.942	3	0.537
Appendix Supp. Fig S3B-CD68	P4 (2.0E12) Single	1.433	0.323	0.936	3	0.510
Appendix Supp. Fig S3B-CD68	P4 (4.0E12) Single	1.833	0.666	0.812	3	0.144
Appendix Supp. Fig S3B-MRC1	WT Untr	1.000	0.283	0.945	2	0.604
Appendix Supp. Fig S3B-MRC1	P4 (1.0E12) Multiple	1.100	0.300	1.000	3	1.000
Appendix Supp. Fig S3B-MRC1	P4 (1.0E12) Single	1.300	0.200	1.000	3	1.000
Appendix Supp. Fig S3B-MRC1	P4 (2.0E12) Single	1.200	0.557	0.976	3	0.702
Appendix Supp. Fig S3B-MRC1	P4 (4.0E12) Single	1.267	0.473	0.907	3	0.409
