# Supplementary material for: Promoterless gene targeting without nucleases rescues lethality of a Crigler‐Najjar syndrome mouse model
Source: EMBO Mol Med. 2017 Jul 27;9(10):1346–55. doi: 10.15252/emmm.201707601 (PMC5623861; doi:10.15252/emmm.201707601)
Supplement: Supplementary file 2 — Expanded View Figures PDF [file EMMM-9-1346-s002.pdf]

# Expanded View Figures

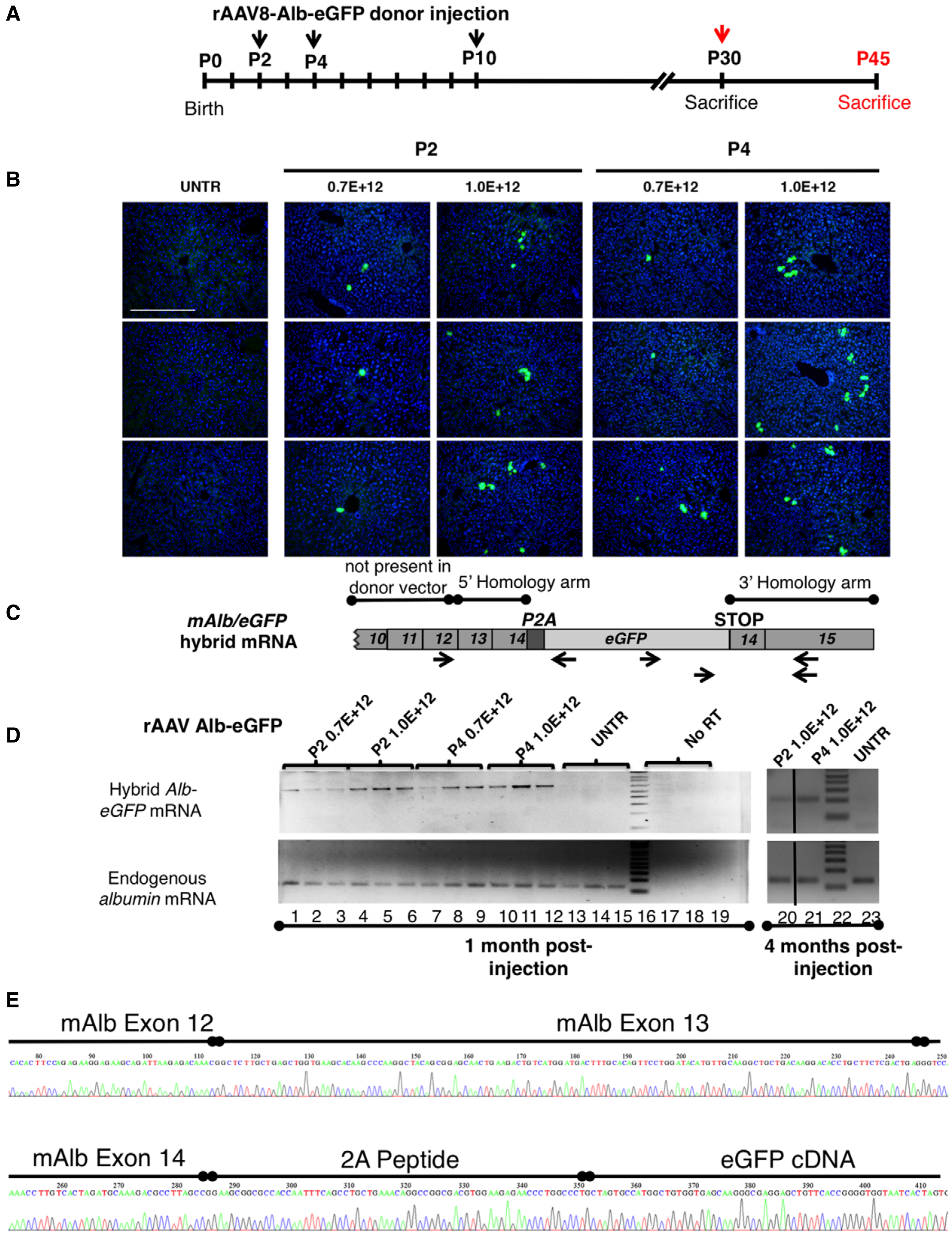


**Figure EV1.**

© 2017 International Centre for Genetic Engineering and Biotechnology

◀ **Figure EV1. Transduction of WT mice with the rAAV8-Alb-eGFP donor vector. Experimental scheme and molecular analysis.**

A Experimental scheme. P2, P4, and P10 WT mice were IP transduced with rAAV8-Alb-eGFP at two doses (0.7E12 and 1.0E12 vgp/mouse) and sacrificed at P30. P30 WT mice were i.v. transduced and sacrificed at P45 with 1E12 vgp/mouse (Fig 1B–D).

B Histological analysis of liver sections from WT neonate mice treated with rAAV-Alb-eGFP. Nuclei were counterstained with Hoechst (blue signal). Each field corresponds to a single animal ($n$ = 3 per time point/treatment). Scale bar, 200 μm.

C Scheme of the targeted cDNA indicating the position of the regions of homology, P2A, eGFP cDNA, the position of the primers used for the RT–PCR shown in the agarose gels (panel D) and for the amplification of the sequenced fragment (panel E).

D Semi-quantitative RT–PCR of liver total RNA samples from WT mice injected at P2 or P4 with AAV-Alb-eGFP, as shown in panel (B). Animals were sacrificed at 1 and 4 months post-injection (lanes 1–15, and 20, 21, and 23, respectively). The primers annealed to the eGFP cDNA and to the albumin exon 15. A 1,123-base intron is present between albumin exons 14 and 15. The endogenous albumin mRNA was used as control. Lanes 16 and 22, molecular weight markers.

E Molecular analysis of the chimeric mRNAs. Chromatogram obtained by sequencing the chimeric Albumin-2A-eGFP cDNA RT–PCR product. The RT–PCR products obtained with the primers indicated in panel (C) were cloned in pGEM-T vector and sequenced. The mouse albumin exons 12, 13, and 14 are indicated, as well as the peptide 2A and the human eGFP cDNA.

 © 2017 International Centre for Genetic Engineering and Biotechnology

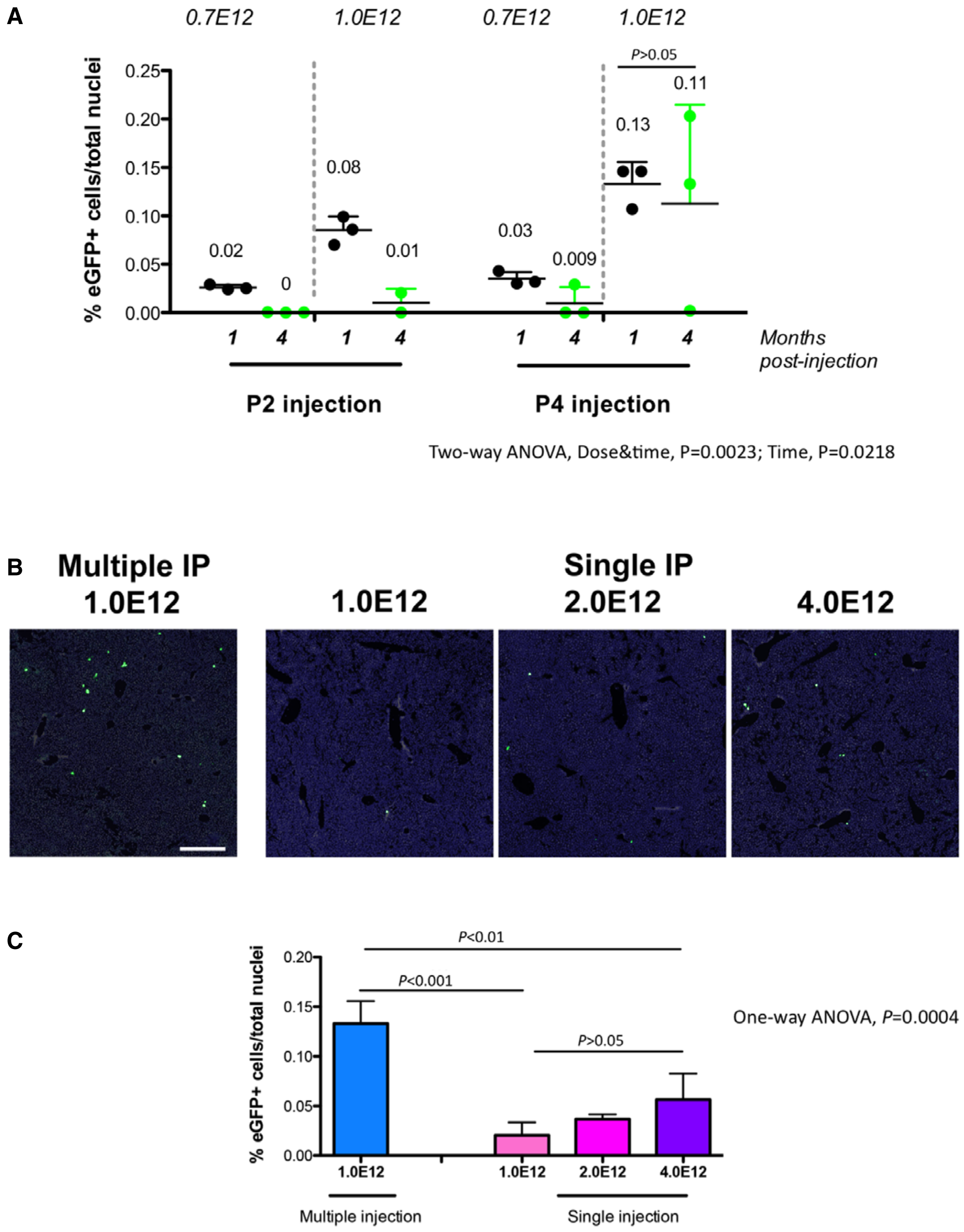


**Figure EV2. Transduction of WT neonate mice with the AAV-Alb-eGFP donor vector with different doses, injection age, modality, and analysis time points.**

A P2 and P4 WT pups were IP transduced with rAAV-Alb-eGFP at two doses: 0.7E12 and 1.0E12 vgp/mouse. The % of eGFP-positive cells at 1 and 4 months is indicated. Each single dot represents an animal. Three sections per animals were analyzed. Results are expressed as mean ± SD. The numbers above each group indicate the mean value. Two-way ANOVA: interaction, NS; treatment, $P = 0.0023$; time, $P = 0.0218$; Bonferroni *post hoc* tests: time: P4 1.0E12 1 month versus P4 1.0E12 4 months, $t = 0.6343$, $P =$ ns.

B, C Comparison of multiple and single AAV-Alb-eGFP administration of WT P4 neonates at different doses by IP injection. $N = 3$ per time point/dose. Three sections per animals were analyzed. One-way ANOVA: $F = 21.20$, $P = 0.0004$; Bonferroni comparison tests: multiple vs. 1.0E12, 2.0E12, 4.0E12, $t = 7.360$, $t = 6.293$, $t = 5.008$, $P < 0.001$, $P < 0.01$, $P < 0.01$, respectively; 1.0E12 versus 2.0E12, $t = 1.067$, 1.0E12 versus 4.0E12, $t = 2.352$, 2.0E12 versus 4.0E12, $t = 1.285$, all $P =$ ns. Results are expressed as mean ± SD. Scale bar, 400 μm.

© 2017 International Centre for Genetic Engineering and Biotechnology

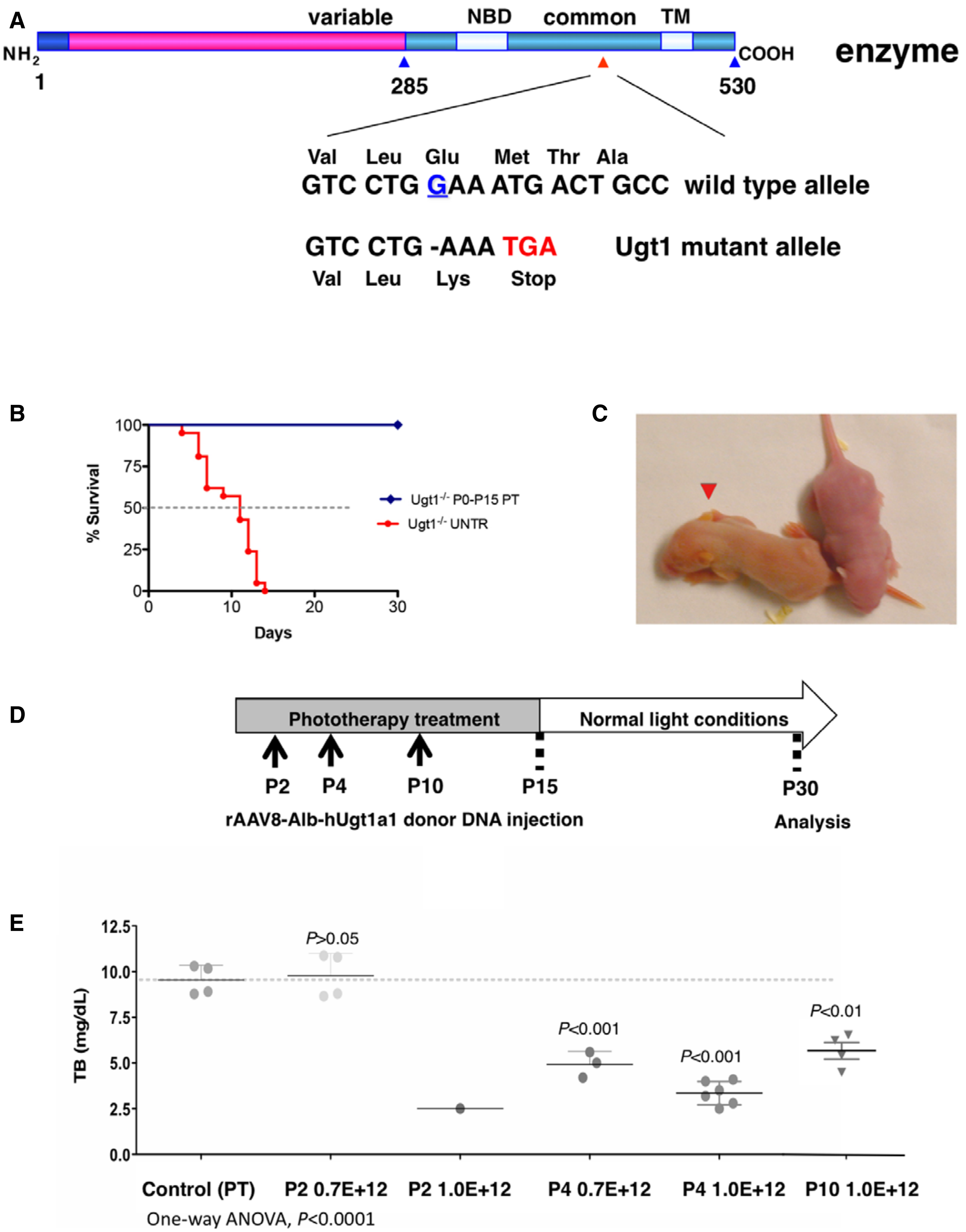


**Figure EV3. The GeneRide approach in the Ugt1$^{-/-}$ mouse strain.**

- A Scheme of the protein with the position of the targeted one-base deletion and the DNA and protein sequences of the WT and mutant alleles.
- B Kaplan–Meier survival curve of WT and Ugt1$^{-/-}$ mutant mice.
- C The red triangle indicates the mutant pup. Its orange discoloration caused by severe hyperbilirubinemia is evident 36 h after birth. Panels (B and C) are from Bortolussi *et al* (2014a).
- D, E Transduction of Ugt1$^{-/-}$ mice in non-lethal conditions with the rAAV-Alb-hUgt1a1 donor vector. Experimental strategy (D). Neonate mutant mice received PT since birth till P15 and were then kept under normal light conditions, were injected at the indicated time points and sacrificed at P30. Plasma bilirubin levels were determined at P30 (E). Controls correspond to Ugt1$^{-/-}$ mice treated only with PT up to P15. Each single dot represents an animal, determined in duplicate (one-way ANOVA: $F = 38.00$, $P < 0.0001$; Bonferroni comparison tests: P4 0.7E12, P4 1.0E12, P10 1.0E12 vs. control, $t = 6.139$, $t = 9.761$, $t = 4.408$, $P < 0.001$, $P < 0.001$, $P < 0.01$, respectively). Results are expressed as mean ± SD.

 © 2017 International Centre for Genetic Engineering and Biotechnology

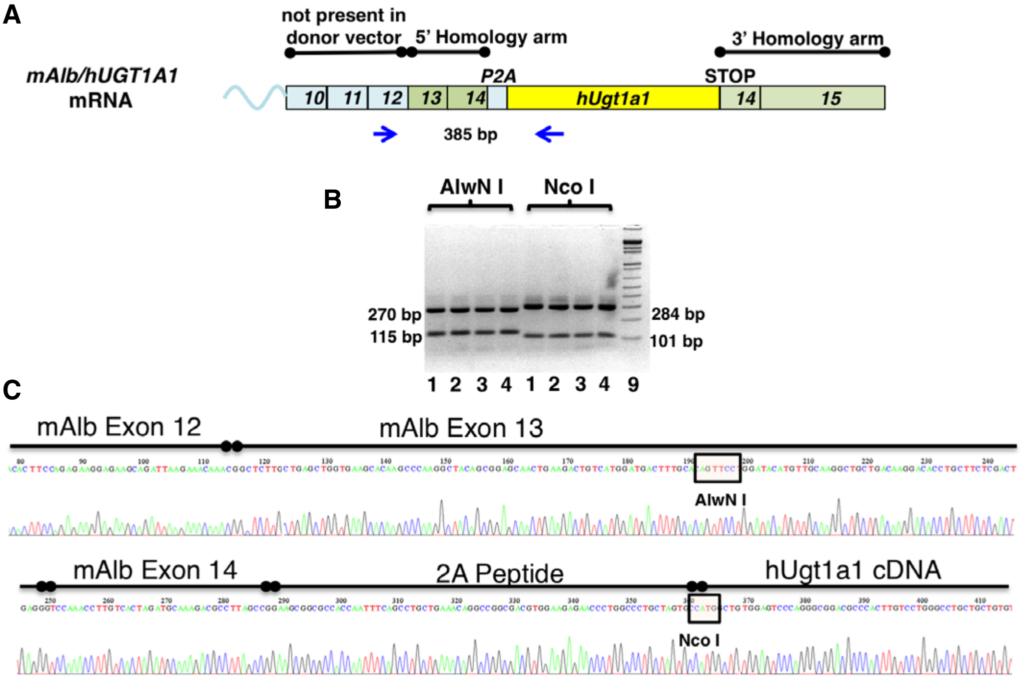


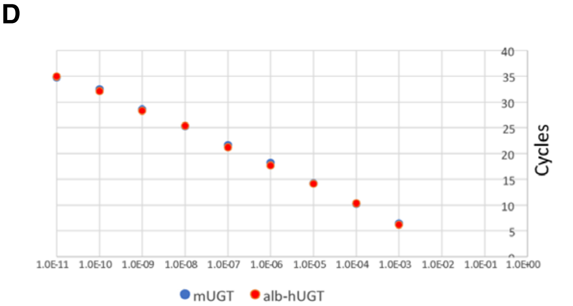


E

| | mUGT | alb-hUGT |
|---|---|---|
| 1,00E-03 | 6,43 | 6,2 |
| 1,00E-04 | 10,26 | 10,31 |
| 1,00E-05 | 14,26 | 14,12 |
| 1,00E-06 | 18,25 | 17,67 |
| 1,00E-07 | 21,71 | 21,2 |
| 1,00E-08 | 25,29 | 25,4 |
| 1,00E-09 | 28,54 | 28,31 |
| 1,00E-10 | 32,47 | 32,14 |
| 1,00E-11 | 34,75 | 34,97 |

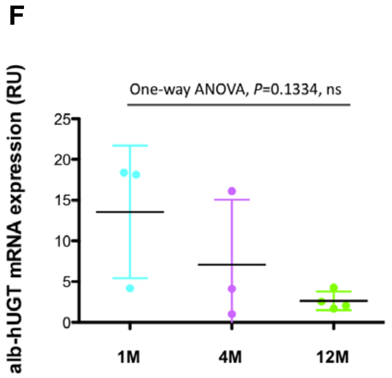


**Figure EV4. Molecular analysis of the GeneRide approach in the Ugt1$^{-/-}$ mouse strain.**

- A Scheme of the chimeric cDNA indicating the regions of homology, P2A, hUgt1a1 cDNA and the position of the primers used for the PCR and the size of the PCR products.
- B The RT–PCR products of liver total RNA samples from mutant mice shown in Fig 3A were digested with AlwNI and NcoI. The size of the fragments is indicated.
- C Chromatogram obtained by sequencing the chimeric Albumin-2A-hUgt1a1 cDNA RT–PCR product. The RT–PCR product obtained with the primers indicated in Fig EV5A was cloned in pGEM-T vector and sequenced. The mouse albumin exons 12, 13, and 14 are indicated, as well as the peptide 2A and the human hUgt1a1 cDNA. The restriction sites AlwNI and NcoI, used to confirm the nature of the RT–PCR product, are indicated.
- D, E Comparison of the efficiency of the PCR primers in the amplification of Alb-hUgt1a1 and mouse Ugt1a1 (mUgt1a1) cDNAs. A set of primers specific for the chimeric Alb-hUgt1a1 mRNA and another one specific for the mouse endogenous Ugt1a1 mRNA were tested in parallel using serial 1:10 dilutions of the same amount of the specific purified DNA templates. The curves are shown in panel (D), while data are shown in panel (E). These conditions and primers were then used to compare the levels of the chimeric Alb-hUgt1a1 mRNA in treated mice with the endogenous mUgt1a1 mRNA levels present in WT mice (Fig 3C).
- F Determination of the levels of the chimeric Alb-hUGT1a1 mRNA in liver of 1-, 4-, and 12-month-old mice. Mice were treated at P4 with 1.0E12 vgp/mouse of rAAV-Alb-hUgt1a1 donor vector. At the indicated time points, mice were sacrificed and levels of the chimeric mRNA analyzed by qRT–PCR. The graph indicates relative levels normalized to albumin mRNA. One-way ANOVA: $F$ = 2.724, $P$ = 0.1334, NS.

© 2017 International Centre for Genetic Engineering and Biotechnology

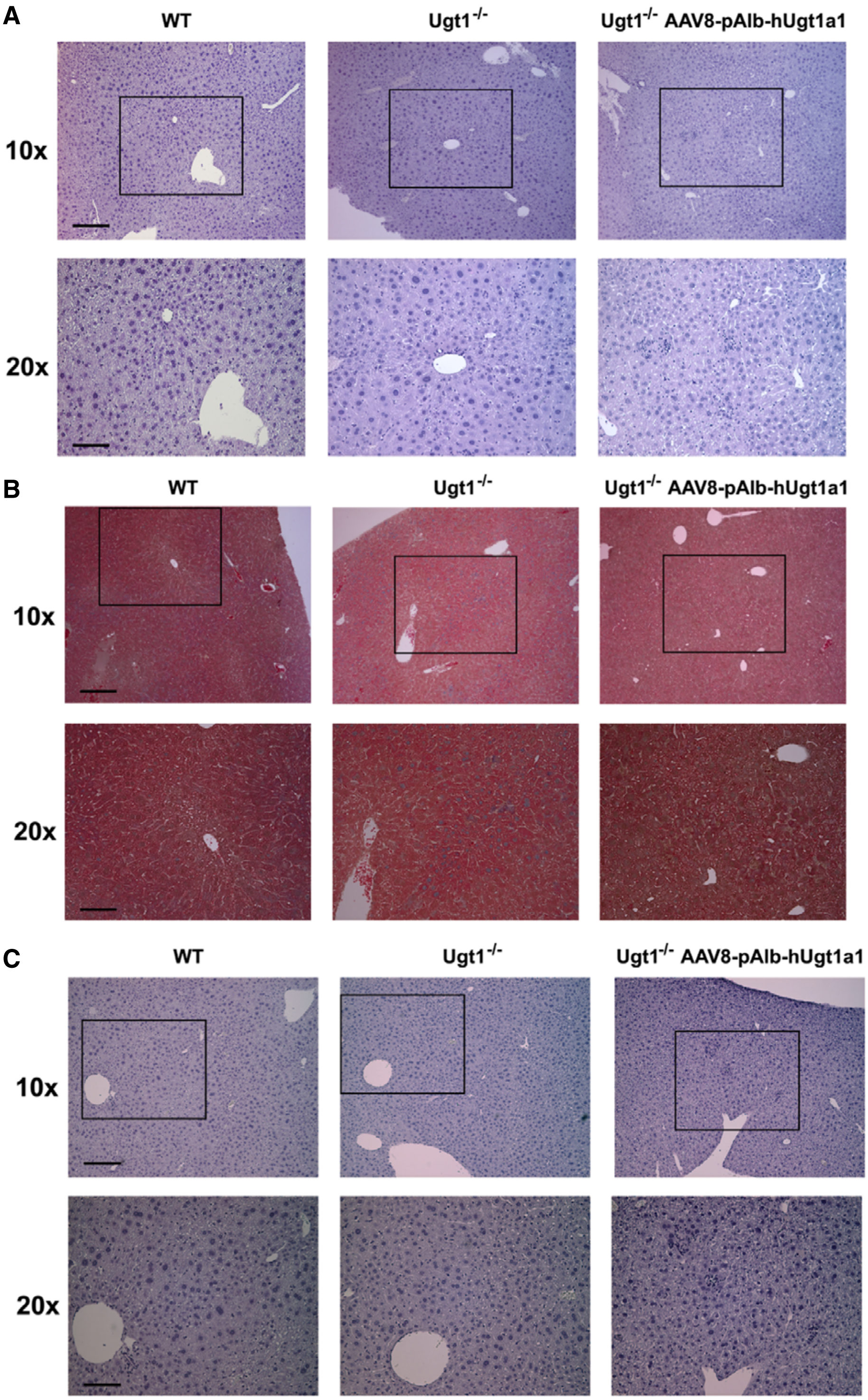


**Figure EV5. Histological analysis of Ugt1$^{-/-}$ mice treated with the rAAV-Alb-hUgt1a1 donor vector.**

A Ugt1$^{-/-}$ mutant mice injected at P4 with 1.0E12 vgp/mouse of rAAV-Alb-hUgt1a1 donor vector were sacrificed at 12 months after viral transfer. Liver sections were stained with hematoxylin–eosin. Representative images are shown. Scale bars, 10×, 200 μm, 20×, 100 μm.

B, C Livers from Ugt1$^{-/-}$ mice treated with the rAAV-Alb-hUgt1a1 donor vector show no evidence of fibrosis or fatty liver. Ugt1$^{-/-}$ mice injected at P4 with 1.0E12 vgp/mouse of rAAV-Alb-hUgt1a1 donor vector were sacrificed at 12 months after viral transfer. Liver sections were stained with Masson's trichrome and Red Oil O staining to detect fibrosis and fatty liver, respectively (panels B and C, respectively). No signs of fibrosis or fatty liver were detected. Representative images are shown. Scale bars, 10×, 200 μm, 20×, 100 μm.

 © 2017 International Centre for Genetic Engineering and Biotechnology
